# Detection of circulating cell-free HPV DNA of 13 HPV types for patients with cervical cancer as potential biomarker to monitor therapy response and to detect relapse

**DOI:** 10.1038/s41416-023-02233-x

**Published:** 2023-03-27

**Authors:** Suzana Mittelstadt, Olga Kelemen, Jakob Admard, Axel Gschwind, André Koch, Sarah Wörz, Ernst Oberlechner, Tobias Engler, Irina Bonzheim, Annette Staebler, Nicola Weidner, Frank Stubenrauch, Thomas Iftner, Olaf Riess, Christopher Schroeder, Stefan Kommoss, Stephan Ossowski

**Affiliations:** 1 Department of Women’s Health, University Hospital Tübingen, Tübingen, Germany; 2 Institute of Medical Genetics and Applied Genomics, University of Tübingen, Tübingen, Germany; 3 NGS Competence Center Tübingen (NCCT), University of Tübingen, Tübingen, Germany; 4 Institute of Pathology and Neuropathology, University Hospital Tübingen, Tübingen, Germany; 5 Department of Radiooncology, University Hospital Tübingen, Tübingen, Germany; 6 Institute for Medical Virology and Epidemiology of Viral Disease, University Hospital Tübingen, Tübingen, Germany; 7 German Cancer Consortium (DKTK), partner site Tübingen and German Cancer Research Center (DKFZ), Heidelberg, Germany; 8 Institute for Bioinformatics and Medical Informatics (IBMI), University of Tübingen, Tübingen, Germany

**Keywords:** Cervical cancer, Prognostic markers, Diagnostic markers

## Abstract

**Background:**

HPV-related cervical cancer (CC) is the fourth most frequent cancer in women worldwide. Cell-free tumour DNA is a potent biomarker to detect treatment response, residual disease, and relapse. We investigated the potential use of cell-free circulating HPV-DNA (cfHPV-DNA) in plasma of patients with CC.

**Methods:**

cfHPV-DNA levels were measured using a highly sensitive next-generation sequencing-based approach targeting a panel of 13 high-risk HPV types.

**Results:**

Sequencing was performed in 69 blood samples collected from 35 patients, of which 26 were treatment-naive when the first liquid biopsy sample was retrieved. cfHPV-DNA was successfully detected in 22/26 (85%) cases. A significant correlation between tumour burden and cfHPV-DNA levels was observed: cfHPV-DNA was detectable in all treatment-naive patients with advanced-stage disease (17/17, FIGO IB3–IVB) and in 5/9 patients with early-stage disease (FIGO IA–IB2). Sequential samples revealed a decrease of cfHPV-DNA levels in 7 patients corresponding treatment response and an increase in a patient with relapse.

**Conclusions:**

In this proof-of-concept study we demonstrated the potential of cfHPV-DNA as a biomarker for therapy monitoring in patients with primary and recurrent CC. Our findings facilitate the development of a sensitive and precise, non-invasive, inexpensive, and easily accessible tool in CC diagnosis, therapy monitoring and follow-up.

## Introduction

Cervical cancer is the most prevalent human papilloma virus (HPV)-associated cancer and the fourth most common cancer in women [1]. Ninety-nine percent of cervical cancer cases are associated with HPV infections [2, 3]. Prognosis and response to treatment are generally good when cervical cancer is diagnosed at an early stage (stage IA to IB2—FIGO 2018) with a 5-year survival rate of at least 92% [4]. For women diagnosed at advanced stages (stage IB3–IVB—FIGO 2018), the prognosis is poor with higher recurrence rates and worse survival: when lymph nodes are affected (stage IIIC) the 5 years survival is 59% and when metastatic disease is found (stage IVA to IVB—FIGO 2018) just 17%. It is estimated that at least 30% of patients in advanced stages will relapse [5]. Early-stage cervical cancer is usually treated with surgery, while locally advanced stages are typically treated with chemoradiation [6]. Metastatic disease is treated with palliative chemotherapy, often combined with radiation for treatment of symptoms such as bleeding or pelvic pain. Current guidelines suggest a patient follow-up and therapy monitoring based on clinical examination and, when indicated, cross-sectional imaging [7].

Infections with HPV, which is a large group of more than 200 different DNA viruses, normally resolve spontaneously, but in some cases persist resulting in warts or precancerous lesions [8]. Persistent infection can be accompanied by an increasing number of viral episomes and the integration of the viral genome into the host genome. Both mechanisms are thought to drive genomic instability and initiate tumour development [9]. Different HPV types can be categorised according to their carcinogenic potential. The Working Group of the World Health Organisation International Agency for Research on Cancer (IARC) classifies the HPV types 16, 18, 31, 33, 35, 39, 45, 51, 52, 56, 58, 59 as group I carcinogens (potential carcinogenic) and HPV 68 as group IIA (probably carcinogenic) [10]. Of them, the most prevalent ones are HPV16 and HPV18, the first one is responsible for over 50% of cervical cancers and the second one for approximately 15% [10].

Liquid biopsies are minimally invasive samples of urine, saliva or blood that can be examined for cell-free DNA (cfDNA). Cell-free circulating tumour DNA in plasma is a well-known biomarker for monitoring treatment response and detection of residual disease or relapse of cancer patients [11]. Most published methods aim for detecting oncogenic and other somatic mutations or cancer-specific DNA methylation patterns in the cfDNA [12]. Cell-free circulating HPV-DNA (cfHPV-DNA) is another detectable tumour-specific DNA that can be easily distinguished from human DNA [13]. First studies using ddPCR detected HPV16, HPV18 and HPV 45 DNA in plasma and the results indicate that liquid biopsies can be used for treatment monitoring and detection of minimal residual disease (MRD) [14–18].

Technological advances have helped to significantly improve the sensitivity of cfDNA tests. The aim of this proof-of-concept study was to assess a highly sensitive targeted next-generation sequencing (NGS) approach to simultaneously detect multiple high-risk HPV types in plasma of cervical cancer patients and evaluate its correlation with disease parameters such as stage and treatment response to comprehensively investigate its potential as biomarker for cervical cancer management.

## Materials and Methods

### Patient cohort and biobank

Plasma samples from the biobank of the University Women’s Hospital Tübingen collected between 2016 and 2021 were screened for patients with histological confirmed cervical cancer. All patients provided written informed consent in accordance with institutional and federal guidelines for the collection and storage of their samples in the biobank. Clinical data were obtained from chart review. The study was approved by the ethic commission of the University Tübingen Hospital (project ID: 946/2020BO2). Samples were collected as 10 mL peripheral whole blood in cell-stabilisation blood collection tubes (Cell-free DNA BCT tubes, Streck, Omaha, USA) or as 9 mL in EDTA tubes without preparation. EDTA samples were centrifuged within 2 h after the samples were obtained, cell-stabilisation blood collection tubes were processed in accordance with the manufacturer’s recommendations. Plasma was separated from blood using a double centrifugation protocol (1900 × *g* for 10 min at RT). Separated plasma was stored at −80 °C until DNA extraction was conducted. cfDNA was extracted from plasma using the QIAamp MinElute ccfDNA Kit (Qiagen, Hilden, Germany) according to the manufacturer’s instructions. DNA was eluted twice through the column to maximise yield. Extracted cfDNA was stored at −20 °C until analysis took place.

Corresponding paraffin embedded tumour samples were identified in the archives of the Institute of Pathology at the University of Tübingen, and representative tumour blocks identified by review of the available H&E sections were selected and sequenced. Five-μm unstained slides were obtained from the FFPE blocks. Experienced pathologists assessed tumour content and cellularity and suitable areas of tumour were marked for macro-dissection on the corresponding H&E slide, if necessary. Genomic DNA was extracted from the sections using the Maxwell® RSC DNA FFPE Kit and the Maxwell® RSC Instrument (Promega, Madison, WI, USA) according to the manufacturer’s instructions.

### Next-Generation Sequencing and bioinformatic data analysis

To detect cfHPV-DNA a custom enrichment panel was designed (Twist Bioscience). The panel comprised of the E6 and E7 regions of the viral DNA genome from 13 high-risk HPV types, and 14 short regions of the human genome (˜200 bp each) harbouring common human germline SNVs for verification of sample identity. 12 of the selected HPV types are categorised in IARC group 1 (carcinogenic to humans) i.e., HPV16, HPV18, HPV31, HPV33, HPV35, HPV39, HPV45, HPV51, HPV52, HPV56, HPV58, HPV59, and one type, HPV68, is classified as probably carcinogenic to humans (group 2A). cfDNA was quantified using the Qubit dsDNA HS Assay kit (ThermoFisher Scientific). The quality of cfDNA was evaluated with an Agilent 2200 TapeStation using High Sensitivity D1000 ScreenTape Assay (Agilent). Libraries were prepared using the Twist Library Preparation Kit (Twist Bioscience) with xGen UDI-UMI adaptors (IDT) and the Twist Target Enrichment Kit. Batches of 8 libraries were combined and multiplexed in the capture reaction. Enriched plexes were equally pooled for sequencing. Libraries were sequenced on the Illumina NovaSeq6000 platform (Illumina, San Diego, CA, USA) in paired-end mode (PE, 2×100bp). Additional read-out for fragment unique molecular identifiers (UMI, 9 bases) was generated. Libraries were sequenced at an average depth of 31 million reads.

Quality control and processing of raw data from DNA libraries was performed using the in-house megSAP pipeline megSAP (https://github.com/imgag/megSAP, version 0.2–266-gb6e434f) and the ngs-bits package (https://github.com/imgag/ngs-bits, version 2020_03), as described before [19, 20]. Briefly, sequencing reads were aligned using BWA-MEM (version 0.7.17) to a combined reference including the human reference genome (GRCh37) and viral genomes for all HPV types included in the panel design. Aligned fragments were deduplicated and error-corrected using umiVar2 (unpublished tool for UMI-based sequencing-error correction) based on the corresponding UMI bases and alignment coordinates. Abundance of viral sequence content was then obtained using high-quality alignments only and by calculating average per-base coverage for E6 and E7 regions. Viral coverage was normalised with mean coverage across enriched human genome regions.

The patients’ samples were separated into two groups: the first one, called treatment-naive, consisted of samples collected before any kind of therapeutic intervention (i.e., surgery as conization or hysterectomy, radiation or chemotherapy—diagnostic procedures such as biopsies or curettage were not considered as therapeutic intervention); and the second group, non-treatment-naive, was composed of samples that were collected after a therapeutic intervention. If a sample was collected at relapse, it was considered treatment-naive if it was collected before the therapy for relapse and non-treatment-naive if was collected after the beginning of the relapse therapy.

Data were visualised using Python (version 3.9.7) and the matplotlib library (version 3.4.3). All statistical analyses and visualisation were performed using the R Statistical Software [21], the *dplyr* [22], and the *ggplot2* [23] packages. For the graphic representations, the levels of cfHPV-DNA were assumed as being the highest reads between E6 and E7 for all HPV types, and when the levels were more than 10,000 reads, a logarithmic scale (The base 10 logarithm of *x* + 1 was used to deal with the observations in which *x* = 0) was used for better visualisation.

## Results

### Patient cohort

**Fig. 1:**
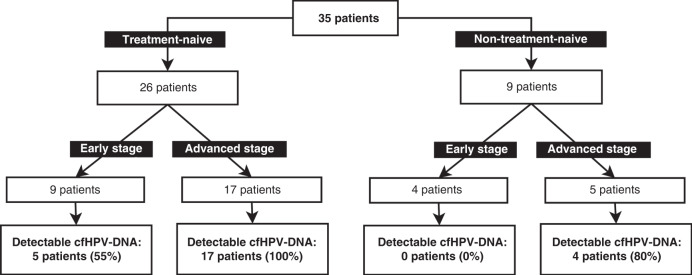
Study design. Treatment-naive group include cases with available samples before treatment. Non-treatment-naive group is composed of patients with available samples only after any therapeutic intervention.

Samples of a total of 35 patients with cervical cancer were included (Fig. 1). The mean age at diagnosis was 46.1 years (SD 12.9 years). Histological type was squamous cell carcinoma for 71% of patients, followed by adenocarcinoma for 23%, and adenosquamous for 6% (Table 1). Most patients (62.3%) had advanced stage disease (IB3–IVB—FIGO 2018), corresponding to locally advanced tumours with more than 4 cm (FIGO IB3), with vagina or parametrium infiltration (FIGO II–IIIB), disease which has spread to local lymph nodes (FIGO IIIC), clinically involved bladder and/or rectum mucosa (FIGO IVA) or distant metastasis (FIGO IVB).

**Table 1:** Patient’s characteristics.

		*N* (%)
Mean age		46.1 years
FIGO stage	IA1	5 (14%)
	IB1–2	8 (23%)
	IB3	1 (3%)
	IIA-B	3 (8%)
	IIIB	1 (3%)
	IIIC1–2	13 (37%)
	IVA	2 (6%)
	IVB	2 (6%)
Histology	Squamous carcinoma	25 (71%)
	Adenocarcinoma	8 (23%)
	Adenosquamous carcinoma	2 (6%)

### Liquid biopsies

**Fig. 2:**
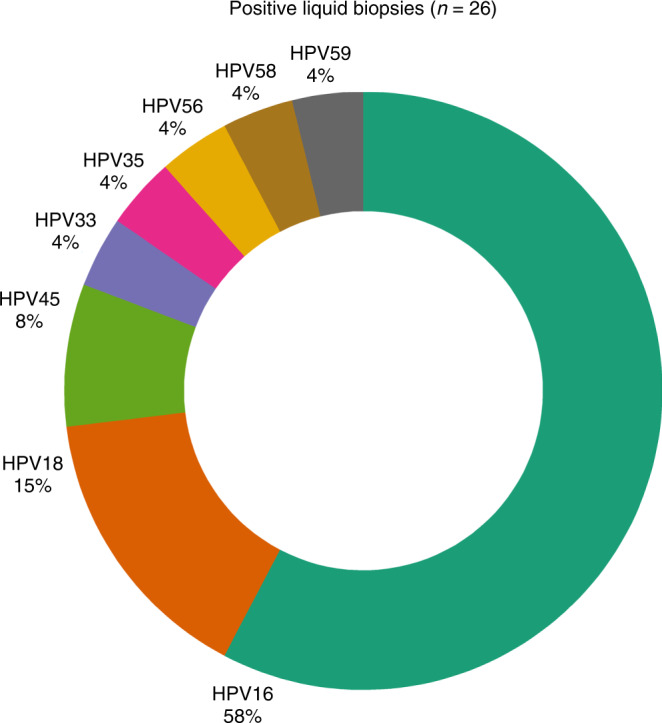
HPV types distribution. Doughnut chart with proportion of HPV types detected in the liquid biopsies.

cfDNA with adequate quality was isolated from 69 samples and sequenced to a median depth of 400,049× (range: 16,809–733,959×, IQR 322,876×). After deduplication of sequencing reads, which provides counts of unique DNA fragments by removing PCR duplicates, a median depth of 8853× (range: 241–23,558×, IQR 4478×) was achieved. The most prevalent HPV types in cfDNA were HPV16 followed by HPV18, other less prevalent types were HPV33, HPV35, HPV45, HPV56, HPV58 and HPV59 (Fig. 2). To evaluate if the cfHPV-DNA found in plasma was the same HPV type present in the tumour, paraffin embedded tumour samples of 9 patients were sequenced and viral DNA was found in all of them (HPV16, HPV18, HPV33, HPV35 and HPV56). For all patients the HPV types detected in plasma matched results from tumour tissue HPV testing, corroborating that the experimental method is plausible. Tumour samples of 2 patients had 2 different HPV types detectable, and for these patients the HPV type found in cfDNA was the dominant one (i.e., the HPV type with more reads in tumour tissue).

Of the 34 treatment-naive samples from 26 patients (some patients had replicate pre-treatment samples), cfHPV-DNA was detectable in 30 (88%): detectable cfHPV-DNA was found in all 23 treatment-naive samples of patients in advanced stage (IB3–IVB); and of the 11 samples of patients in early stage (IA1–IB2), 7/11 (64%) had detectable cfHPV-DNA. From a patient-centred view, 17 of 17 patients in advanced stage had detectable levels of cfHPV-DNA but only in 5 of 9 patients in early stage cfHPV-DNA were detectable.

**Fig. 3:**
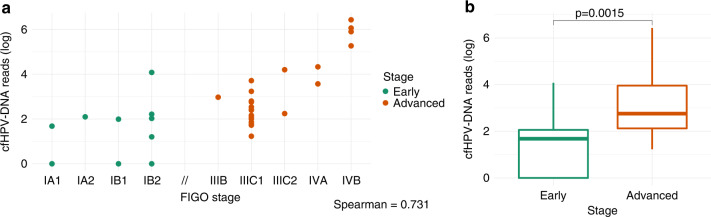
cfHPV-DNA and disease staging. Scatterplot (**a**) and boxplot (**b**) with correlation between cfHPV-DNA levels and disease stage (FIGO 2018); *p* value was calculated with Wilcoxon test.

For 9 patients, no treatment-naive plasma samples were available (see Fig. 1 study design). All of them had at least a conization before the sample was collected. Only 4 of 12 samples (33%) showed detectable cfHPV-DNA, all four were from cases in advanced stages. The only patient in advanced stage with undetectable cfHPV-DNA had a FIGO IIIC1 disease because of one affected lymph node (sentinel node) and the plasma sample was collected after conization and pelvic and paraaortic lymphadenectomy. All patients in early stage presented undetectable cfHPV-DNA in the liquid biopsy, indicating that the amount of cfHPV-DNA may be strongly affected by interventions that affect the tumour burden. This finding coincides with and confirms the observation that cfHPV-DNA levels increase in advanced stages of the disease, i.e., correlate with tumour burden and spread. We observed a moderate correlation, Spearman’s *ρ* = 0.731, between FIGO-Stage and levels of cfHPV-DNA (Fig. 3a, scatterplot) for treatment-naive samples and a highly significant correlation when comparing early and advanced stages (Fig. 3b, Wilcoxon test *p* = 0.0015).

### Therapy monitoring

**Fig. 4:**
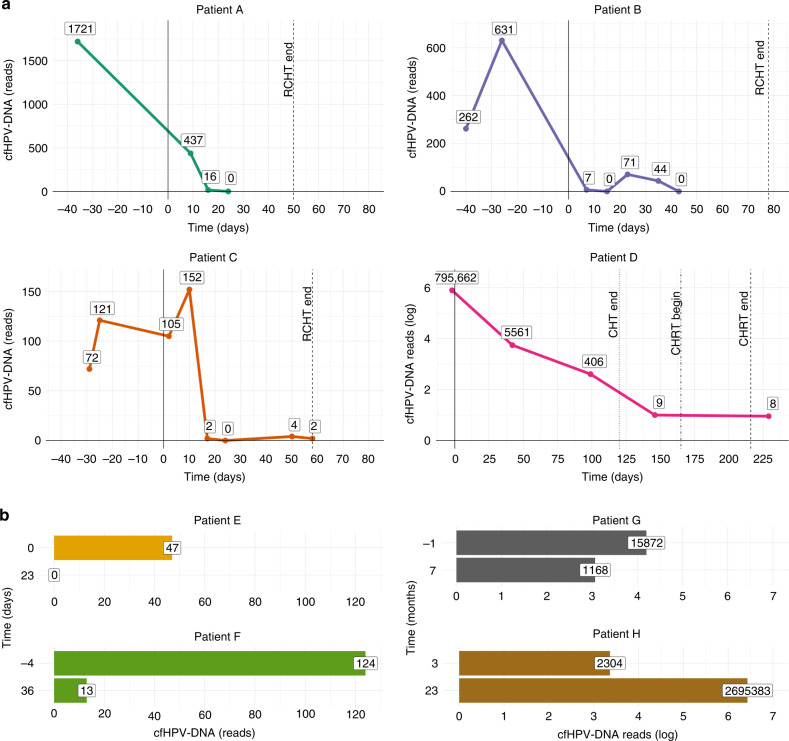
Graphs representing the change in levels of cfHPV-DNA throughout time for 8 patients. Time 0 represents the beginning of the therapy. **a** Patients A–C underwent primary chemoradiation by FIGO IIIC1 disease and presented a complete remission and had at least 1.5-year follow-up without evidence of a relapse. Patient D received primary chemotherapy associated with Bevacizumab followed by chemoradiation and also presented a complete remission, Bevacizumab was continued after the radiotherapy and the follow-up after two years still showed no evidence of disease. **b** For patient E, a conization were performed for FIGO IA1 disease. Patient F had the first sample before a re-conization by local relapse (FIGO IA2 as relapse after 13 months after primary disease with FIGO IA1), and the second sample after one month, because of compromised conization margins a radical hysterectomy was performed 2 days after the second plasma sample. Patient G with FIGO IIIC2 had a pre-treatment sample and other 6 months after the end of primary chemoradiation, this patient suffered a disease relapse one year after the end of the chemoradiation (and 6 months after the second sample). Patient H (FIGO IVA) had no baseline sample available, but one 1 month after the end of chemoradiation, because of a vesicovaginal fistula and extensive necrosis, an anterior pelvic exenteration was performed 2 months after the end of the chemoradiation (i.e., 1 month after the first liquid biopsy), the histopathological examination showed no vital tumour, the second sample was collected one and half years later when disseminate metastases (liver, peritoneal and thoracic lymph nodes) were diagnosed.

Therapy monitoring was performed for 8 patients by analysing sequential samples taken in different points of treatment. For two patients with early-stage disease (FIGO IA), we analysed one sample before and one after conization. The comparison of cfHPV-DNA levels showed a substantial decrease after surgery. For one patient (Fig. 4b, patient E), the second sample taken 23 days after conization showed no detectable cfHPV-DNA reads anymore. For the other patient (Fig. 4b, patient F) the second sample was collected 36 days after the conization and showed decreasing but still detectable cfHPV-DNA levels. The conization of these patients revealed compromised surgical margins and a radical hysterectomy was performed 2 days after the second plasma sample. The histopathological examination of the uterus showed 5 mm residual tumour, hence explaining the residual disease detected in plasma.

Three patients with advanced stage disease (FIGO IIIC1) under primary chemoradiation had serial samples (4–8 samples per patient). A quick decrease of cfHPV-DNA under chemoradiation was noted, which reflected their clinical response to treatment. Undetectable levels of cfHPV-DNA in plasma were generally reached within 1 month of treatment (Fig. 4a, patients A–C), with a rapid decline already within the first 20 days. One patient (Fig. 4a, patient B) showed a small peak between days 20 and 35, after showing undetectable levels at day 15. All three patients had at least 1.5-year follow-up without evidence of a relapse. For a fourth patient (Fig. 4b, patient G) with advanced cervical cancer (FIGO IIIC2) we had, in addition to the pre-treatment sample, only one liquid biopsy taken 6 months after the end of primary chemoradiation. Although the cfHPV-DNA levels decreased in comparison to the first sample it was still relatively high, indicating a potential relapse. Indeed, this patient suffered a disease relapse one year after the end of the chemoradiation (and 6 months after the second sample). This indicates that cfHPV-DNA based liquid biopsy could be a valuable tool for early detection of relapse and more generally for detection of minimal residual disease. For a fifth patient (Fig. 4b, patient H) with advanced disease (FIGO IVA—bladder infiltration) we had no baseline (pre-treatment) sample available. However, the first liquid biopsy collected one month after the end of chemoradiation still showed a high level of cfHPV-DNA. Because of a vesicovaginal fistula and extensive necrosis, an anterior pelvic exenteration was performed 2 months after the end of the chemoradiation (i.e., 1 month after the first liquid biopsy). In the histopathological examination no vital tumour was found. A second liquid biopsy sample was collected one and half years later when disseminate metastases (liver, peritoneal and thoracic lymph nodes) were diagnosed. In this sample we observed an exponential increase of cfHPV-DNA levels compared to the post-chemoradiation sample.

Finally, one patient (Fig. 4a, patient D) with primary metastatic disease (FIGO IVB—supraclavicular lymph node), received primary chemotherapy associated with Bevacizumab and, subsequently, radiotherapy. The patient had a complete remission accompanied by a quick decrease of cfHPV-DNA observed under therapy. Bevacizumab was continued after the radiotherapy and the follow-up after 2 years still showed no evidence of disease. The patient had an extremely high load of cfHPV-DNA (795.662 sequenced reads) pre-treatment, that dropped within 30 days of chemotherapy to less than 1% and after 100 days to less than 0.1% of the original level. Interestingly, we could still detect cfHPV-DNA after the end of radiotherapy, with only 8 remaining reads, indicating that the continued Bevacizumab therapy might have been essential for the absence of a relapse after 2 years.

## Discussion

Cell-free tumour DNA is a minimal invasive biomarker that holds potential to be used for monitoring of treatment response and to detect minimal residual disease post-treatment in cancer patients. Early studies used PCR and qPCR approaches to detect cfHPV-DNA in plasma of cervical cancer patients finding HPV16/HPV18 DNA in 20–30% of samples [24–27]. More sensitive methods such as ddPCR found 31%-62% cfHPV-DNA positive samples [15, 16]. An important factor that affects the detection rate (sensitivity) is tumour stage, and the detection rate can be up to 100% for patients with metastatic cervical cancer [17]. In this study we found that levels of cfHPV-DNA are highly correlated with FIGO-stage and reached a detection rate of 100% for FIGO IB3-IVB.

Another important aspect is the detection of different HPV types. A previous study using a broader range of HPV types was able to increase the rate of HPV detection in cervical cancer [28]. While most of the previous studies with cfHPV-DNA focused on HPV16 and 18, the NGS approach in this study was designed to detect a broader range of 13 high-risk HPV types [15, 24–26]. Besides HPV16 and HPV18, we indeed found several other oncogenic HPV types including HPV33, HPV35, HPV45, HPV56, HPV58 and HPV59 (Fig. 2). If in the present study we would have only analysed HPV16/HPV18, the detection rate would have been comparable to previously described studies with 61% (16/26). Compared to a more comprehensive cfHPV-test that detects 5 types of HPV and is clinically validated for oropharyngeal cancer [29], our test increases the sensitivity by over 19% percentage points by detecting a wider spectrum of HPV types. The overall detection rate of our method including the additional HPV types reached 85% (22/26) for treatment-naive patients. This illustrates that a broader panel of HPV types can increase the detection rate and thus the value of cfDNA-HPV as a biomarker itself.

Previous studies showed that presence of viral cfDNA before treatment is associated with an increased risk of disease recurrence and death. Monitoring of viral cfDNA was shown to correlate with treatment response in a small study with an experimental therapy [16, 17]. In the present study, we observed in seven patients that decreasing levels of cfHPV-DNA fraction under treatment correspond to the clinical therapeutic response (remission), and that increasing levels in one patient corresponded with relapse. The detection rate of cfHPV-DNA for patients with samples taken before therapy was as high as 85% (22/26). Stratification according to the stage revealed that 100% (17/17) of patients in advanced stage presented detectable cfHPV-DNA while in early stage only 55% (5/9) did. Based on our results with a limited number of patients, we hypothesise that: (1) levels of cfHPV-DNA are correlated with FIGO-stage and consequently with increased risk of recurrence, (2) high levels of cfHPV-DNA pre-treatment can rapidly decrease to (nearly) undetectable levels when a favourable therapy response is achieved, (3) decrease of cfHPV-DNA to nearly undetectable levels (no significant MRD) seems to correlate with better prognosis despite initial stage (4) detectable cfHPV-DNA at high levels post-treatment (significant MRD) can indicate a high risk to relapse even with no evidence of disease in histopathological examination; (5) cfHPV-DNA may decrease quickly with reduction of tumour burden in early stage but low-levels cfHPV-DNA may be associated with residual disease.

Limitations of this study are the small samples size and the heterogeneous sequential sampling protocols (different number and frequency of blood draws) during therapy.

In conclusion, our and previous results indicate that cfHPV-DNA is a potential tumour biomarker to monitor treatment response in cervical cancer patients. Although the comparability of the different studies is limited due to small cohort sizes and differences in cohort composition, the results indicate an important role of the tumour stage. cfHPV-DNA can be detected in about half the cases of early-stage cervical cancer including microscopic disease (FIGO IA1) and is expected to be detectable in most advanced stages. The novel NGS approach used in this study shows a higher detection rate than previous studies due to the inclusion of 13 high-risk HPV types instead of just HPV16/18, which significantly increased the sensitivity up to a 100% for advanced stages. Nonetheless, additional clinical trials are needed to establish definitively the prognostic and potential therapeutic value of cfHPV-DNA in plasma of cervical cancer patients.

## Data Availability

The datasets used and analysed during the current study are available from the corresponding author on reasonable request.

## References

[1] Sung H, Ferlay J, Siegel RL, Laversanne M, Soerjomataram I, Jemal A (2021). Global Cancer Statistics 2020: GLOBOCAN estimates of incidence and mortality worldwide for 36 cancers in 185 countries. CA Cancer J Clin.

[2] Andersson S, Rylander E, Larsson B, Strand A, Silfversvärd C, Wilander E (2001). The role of human papillomavirus in cervical adenocarcinoma carcinogenesis. Eur J Cancer.

[3] Walboomers JM, Jacobs MV, Manos MM, Bosch FX, Kummer JA, Shah KV (1999). Human papillomavirus is a necessary cause of invasive cervical cancer worldwide. J Pathol.

[4] Bhatla N, Aoki D, Sharma DN, Sankaranarayanan R (2018). Cancer of the cervix uteri. Int J Gynecol Obstet.

[5] National Cancer Institute. SEER cancer stat facts: cervical cancer. SEER 17 2012–2018, all races, females by SEER combined summary stage. 2018. https://seer.cancer.gov/statfacts/html/cervix.html. Accessed 31 Jul 2022.

[6] Cibula D, Pötter R, Planchamp F, Avall-Lundqvist E, Fischerova D, Haie Meder C (2018). The European Society of Gynaecological Oncology/European Society for Radiotherapy and Oncology/European Society of Pathology guidelines for the management of patients with cervical cancer. Radiother Oncol.

[7] Marth C, Landoni F, Mahner S, McCormack M, Gonzalez-Martin A, Colombo N (2017). Cervical cancer: ESMO Clinical Practice Guidelines for diagnosis, treatment and follow-up. Ann Oncol.

[8] zur Hausen H. Papillomaviruses in human cancers. Proc Assoc Am Physicians. 1999;111:581–7. 10.1046/j.1525-1381.1999.99723.x 10591087

[9] Moody CA, Laimins LA (2010). Human papillomavirus oncoproteins: pathways to transformation. Nat Rev Cancer.

[10] IARC Working Group on the Evaluation of Carcinogenic Risks to Humans. Biological agents. A review of human carcinogens. Human papillomaviruses. Lyon: IARC; 2012.

[11] Heitzer E, Haque IS, Roberts CES, Speicher MR (2019). Current and future perspectives of liquid biopsies in genomics-driven oncology. Nat Rev Genet.

[12] Liu MC, Oxnard GR, Klein EA, Swanton C, Seiden MV, CCGA Consortium. (2020). Sensitive and specific multi-cancer detection and localization using methylation signatures in cell-free DNA. Ann Oncol.

[13] Reder H, Taferner VF, Wittekindt C, Bräuninger A, Speel E-JM, Gattenlöhner S (2020). Plasma cell-free human papillomavirus oncogene E6 and E7 DNA predicts outcome in oropharyngeal squamous cell carcinoma. J Mol Diagn.

[14] Sivars L, Hellman K, Crona Guterstam Y, Holzhauser S, Nordenskjöld M, Falconer H (2022). Circulating cell-free tumor human papillomavirus DNA is a promising biomarker in cervical cancer. Gynecol Oncol.

[15] Rungkamoltip P, Temisak S, Piboonprai K, Japrung D, Thangsunan P, Chanpanitkitchot S (2021). Rapid and ultrasensitive detection of circulating human papillomavirus E7 cell-free DNA as a cervical cancer biomarker. Exp Biol Med.

[16] Cheung TH, Yim SF, Yu MY, Worley MJ, Fiascone SJ, Chiu RWK (2019). Liquid biopsy of HPV DNA in cervical cancer. J Clin Virol.

[17] Kang Z, Stevanović S, Hinrichs CS, Cao L (2017). Circulating cell-free DNA for metastatic cervical cancer detection, genotyping, and monitoring. Clin Cancer Res.

[18] Lee JY, Garcia-Murillas I, Cutts RJ, de Castro DG, Grove L, Hurley T (2017). Predicting response to radical (chemo)radiotherapy with circulating HPV DNA in locally advanced head and neck squamous carcinoma. Br J Cancer.

[19] Hilke FJ, Muyas F, Admard J, Kootz B, Nann D, Welz S (2020). Dynamics of cell-free tumour DNA correlate with treatment response of head and neck cancer patients receiving radiochemotherapy. Radiother Oncol.

[20] Bitzer M, Spahn S, Babaei S, Horger M, Singer S, Schulze-Osthoff K (2021). Targeting extracellular and juxtamembrane FGFR2 mutations in chemotherapy-refractory cholangiocarcinoma. NPJ Precis Oncol.

[21] R Foundation for Statistical Computing. R: a language and environment for statistical computing. 2013. http://www.R-project.org/. Accessed 7 Aug 2022.

[22] Wickham H, Francois R, Henry L, Muller K. dplyr: A grammar of data manipulation. 2022. https://CRAN.R-project.org/package=dplyr. Accessed 7 Aug 2022.

[23] Wickham H. ggplot2: Elegant graphics for data analysis. New Tork: Springer New York; 2016.

[24] Liu VW, Tsang P, Yip A, Ng TY, Wong LC, Ngan HY (2001). Low incidence of HPV DNA in sera of pretreatment cervical cancer patients. Gynecol Oncol.

[25] Dong SM, Pai SI, Rha S-H, Hildesheim A, Kurman RJ, Schwartz PE (2002). Detection and quantitation of human papillomavirus DNA in the plasma of patients with cervical carcinoma. Cancer Epidemiol Biomark Prev.

[26] Shimada T, Yamaguchi N, Nishida N, Yamasaki K, Miura K, Katamine S (2010). Human papillomavirus DNA in plasma of patients with HPV16 DNA-positive uterine cervical cancer. Jpn J Clin Oncol.

[27] Chatfield-Reed K, Roche VP, Pan Q (2021). cfDNA detection for HPV+ squamous cell carcinomas. Oral Oncol.

[28] Arroyo Mühr LS, Lagheden C, Lei J, Eklund C, Nordqvist Kleppe S, Sparén P (2020). Deep sequencing detects human papillomavirus (HPV) in cervical cancers negative for HPV by PCR. Br J Cancer.

[29] Chera BS, Kumar S, Beaty BT, Marron D, Jefferys S, Green R (2019). Rapid clearance profile of plasma circulating tumor HPV type 16 DNA during chemoradiotherapy correlates with disease control in HPV-associated oropharyngeal cancer. Clin Cancer Res.

[30] Mittelstadt S, Schroeder C, Kelemen O, Admard J, Gschwind A, Koch A, et al. Abstract: Circulating HPV cell-free DNA in cervical cancer. IGCS 2022 Abstracts: Focused Plenary Presentations. 2022;O032/#771:32.

[31] Mittelstadt S, Schroeder C, Kelemen O, Engler T, Admard J, Gschwind A, et al. Abstract: Zervixkarzinom und zirkulierende HPV zellfreie DNA. 2022. https://dggg2022.abstractserver.com/program/#/details/presentations/1204.

[32] Mittelstadt S, Schroeder C, Kelemen O, Engler T, Admard J, Gschwind A (2022). 2022-RA-1048-ESGO circulating HPV cell-free DNA in cervical cancer. Int J Gynecol. Cancer.

